# Transcriptomic and proteomic analysis of putative digestive proteases in the salivary gland and gut of *Empoasca* (Matsumurasca) *onukii* Matsuda

**DOI:** 10.1186/s12864-021-07578-2

**Published:** 2021-04-15

**Authors:** Ensi Shao, Yujuan Song, Yaomin Wang, Yichen Liao, Yufei Luo, Sijun Liu, Xiong Guan, Zhipeng Huang

**Affiliations:** 1 China National Engineering Research Center of JUNCAO Technology, School of Life Science, Fujian Agriculture and Forestry University, 350002 Fuzhou, Fujian PR China; 2 Department of Entomology, Iowa State University, 50011-3222 Ames, Iowa, USA; 3 State Key Laboratory of Ecological Pest Control for Fujian and Taiwan Crops & Key Laboratory of Biopesticide and Chemical Biology (Ministry of Education), College of Plant Protection, Fujian Agriculture and Forestry University, 350002 Fuzhou, Fujian PR China

**Keywords:** Tea green leafhopper, RNA-Seq, Proteomics, Enzymatic activity, Salivary gland, Gut

## Abstract

**Background:**

Infestation by tea green leafhoppers (*Empoasca* (Matsumurasca) *onukii*) can cause a series of biochemical changes in tea leaves. As a typical cell-rupture feeder, *E. onukii* secretes proteases while using its stylet to probe the tender shoots of tea plants (*Camellia sinensis*). This study identified and analyzed proteases expressed specifically in the salivary gland (SG) and gut of *E. onukii* through enzymatic activity assays complemented with an integrated analysis of transcriptomic and proteomic data.

**Results:**

In total, 129 contigs representing seven types of putative proteases were identified. Transcript abundance of digestive proteases and enzymatic activity assays showed that cathepsin B-like protease, cathepsin L-like protease, and serine proteases (trypsin- and chymotrypsin-like protease) were highly abundant in the gut but moderately abundant in the SG. The abundance pattern of digestive proteases in the SG and gut of *E. onukii* differed from that of other hemipterans, including *Nilaparvata lugens*, *Laodelphax striatellus*, *Acyrthosiphum pisum*, *Halyomorpha halys* and *Nephotettix cincticeps*. Phylogenetic analysis showed that aminopeptidase N-like proteins and serine proteases abundant in the SG or gut of hemipterans formed two distinct clusters.

**Conclusions:**

Altogether, this study provides insightful information on the digestive system of *E. onukii*. Compared to five other hemipteran species, we observed different patterns of proteases abundant in the SG and gut of *E. onukii*. These results will be beneficial in understanding the interaction between tea plants and *E. onukii*.

**Supplementary Information:**

The online version contains supplementary material available at 10.1186/s12864-021-07578-2.

## Background

The tea green leafhopper *Empoasca* (Matsumurasca) *onukii* Matsuda (Hemiptera: Cicadellidae) is an important insect pest of the tea plant *Camellia sinensis* (L.) O. Kuntze (Theaceae) in tea-producing countries in Asia [1–3]. *E. onukii* was previously identified as two species, *E. onukii* and *E. vitis*. A recent detailed study of morphometric characteristics suggests that *E. onukii* and *E. vitis* actually belong to the same species [3]. *E. onukii* damages tea plants by piercing tea buds, leaves and tender shoots with a needle-like stylet to suck the sap, which typically results in a syndrome called hopper burn that dramatically reduces the yield and quality of tea leaves [1]. The annual economic losses caused by this insect could reach 50% in China alone [4]. Currently, the management of *E. onukii* infestation relies largely on the application of chemical pesticides. However, chemical pesticides can lead to pesticide resistance and cause detrimental effects to the environment and human health [5–8]. Biological controls of *E. onukii* have also been reported, but they show very limited success [9–11]. Knowledge of the biochemistry and physiology of the ingestion system of *E. onukii* may spur the development of novel management strategies for this serious insect pest.

Phytophagous hemipteran insects have evolved piercing-sucking mouthparts for sap feeding. There are three types of plant sap-feeding behaviors: phytophagous salivary sheath feeding, osmotic pump feeding, and cell rupture feeding [12, 13]. Studies on stylet activity suggest that *E. onukii* is a typical cell-rupture feeder that secretes enzymes into plant tissues through its stylet and then feeds on mesophyll or stem parenchyma cells [1, 14]. Piercing-sucking feeding requires injecting digestive enzymes secreted from the salivary gland (SG) into plants for extraoral digestion. Hence, a detailed investigation into salivary protein compositions would aid in understanding the physiology of hemipteran insect digestive systems and potentially lead to the development of novel strategies to manage these insect pests.

Salivary protein profiles have been studied at both the transcriptomic and proteomic levels for a wide range of hemipteran insects, including stink bugs, aphids, leafhoppers and planthoppers [15–23]. Salivary proteomic profiling has revealed that saliva from hemipteran insects mainly contains various enzyme families, including oxidoreductases, hydrolases, transferases, Ca^2+^-binding proteins and proteases/peptidases [15, 18, 20, 24, 25]. However, most of those early studies identified only protease groups but not protease species due to the lack of genomic information.

Transcriptomic analysis coupled with proteomic profiling is an effective strategy to identify functional proteins in the SG and gut of hemipterans [21, 26]. For example, proteases in the SG and gut of two devastating pentatomid stink bugs, *Halyomorpha halys* and *Nezara viridula*, were investigated to identify proteases highly expressed in each tissue [21, 26]. Furthermore, mapping of SG transcripts of *H. halys* to the protein profiles of watery saliva, which is the saliva secreted into the host plants, revealed 22 abundant digestive proteases. In addition, the majority of these protease transcripts were highly accumulated in the principle salivary gland (PSG) of *H. halys* and *N. vididula*. These results indicated that in both stink bugs, PSGs were the major sources for releasing proteases into the saliva [21].

Although proteases and nucleases participating in extraoral digestive activities have previously been investigated in the SG and gut of some hemipteran insects, knowledge about the composition of proteases in digestive tissues of *E. onukii* is still limited. We previously analyzed transcriptome sequencing data derived from the gut of *E. onukii*, and putative transcripts encoding proteins of digestive proteases, detoxification enzymes and immune response factors were identified [27]. In the present study, we extended our investigation to transcriptomic and proteomic analyses of the SG and gut of *E. onukii* to investigate tissue-specific expression of proteases. In addition, we also compared the abundance of proteases in the SG and gut of several hemipterans to assess common proteases across the species. The results from this study will not only provide information for a further understanding of the interaction between *E. onukii* and tea plants but also assist in the study of aromatic changes in tea plants after infestation with *E. onukii*. Identification and analysis of the digestive proteases in the SG and gut of *E. onukii* will also assist in the development of novel strategies for managing this important pest.

## Results

### Protease activity in the salivary gland and gut of *E. onukii*

**Fig. 1:**
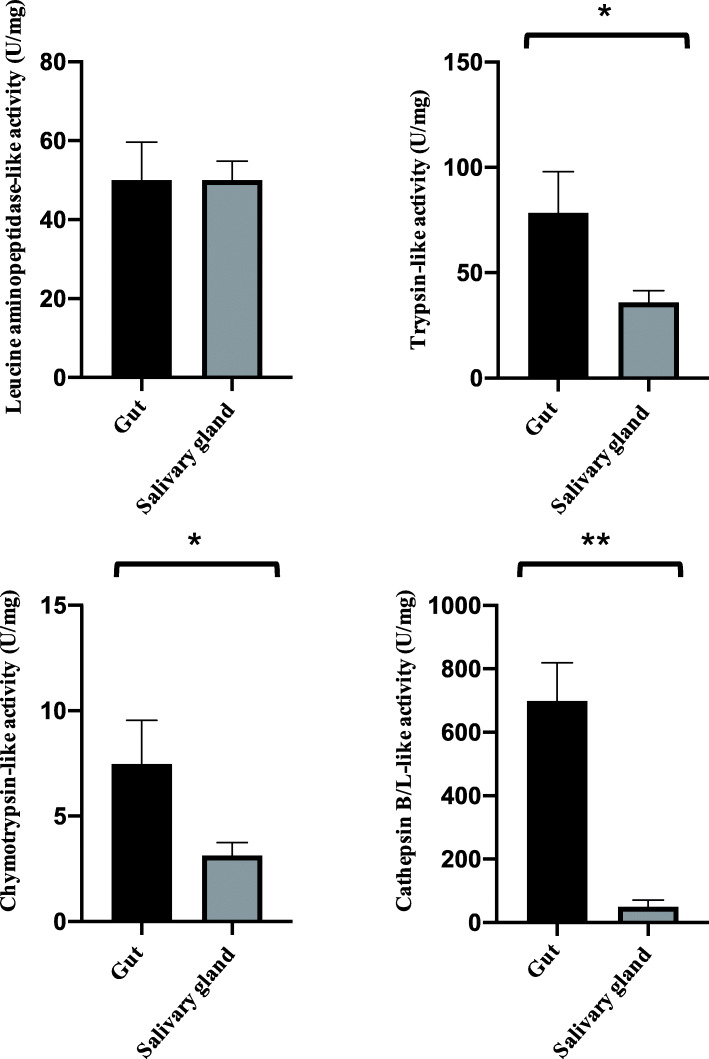
Enzymatic activity of selected proteases in homogenates of salivary glands and guts of *E. onukii*. Error bars indicate the standard deviation from the mean for three replications. Statistical comparisons were conducted between the enzymatic activity of salivary glands and guts (*0.01 < *P <* 0.05, **P < 0.01, Student’s *t*-test)

The enzymatic activities of leucine aminopeptidase, cathepsin B-, cathepsin L-, trypsin- and chymotrypsin-like protease in the SG and gut were examined, and the results are shown in Fig. 1. The overall enzymatic activities of digestive proteases were much higher in the gut than in the SG except for leucine aminopeptidase activities, which showed similar activity units in the SG and gut. The activities of cathepsin B-, cathepsin L-, trypsin- and chymotrypsin-like protease were significantly higher in the gut than in the SG (Fig. 1). Cathepsin B- and L-like proteases showed the highest activities in the gut. Trypsin-like protease, chymotrypsin-like protease and leucine aminopeptidases showed low to moderate activity in the gut (Fig. 1).

### Transcriptomic and proteomic analysis of proteins in the SG and gut of *E. onukii*

1. RNA sequence assembly and functional annotations

The obtained sequence reads were assembled using tissue-specific RNA reads or pooled reads derived from the RNA of both the SG and gut. BUSCO (Benchmarking Universal Single-Copy Orthologs) analysis showed a high-quality (92.1% complete) assembly. Details of the sequence assembly are summarized in Additional file 1: Table S1. The generated contigs were annotated by BLASTx against the NCBI (National Center for Biotechnology Information) nr database. Protein hits were observed for approximately 42, 57 and 38% of the contigs assembled from the SG, gut and pooled reads (Additional file 1: Table S1). The protein hits for the three sets of contigs were similar and reflected in the species distribution of the hits (Additional file 2: Fig. S1). The majority of hits (41–43%) were from hemipterans, followed by ~ 22% from Blattaria (Table 1).

2) Mapping of the assembled genes to proteomic profiles derived from the SG and gut

**Table 1:** Distribution of the top hit annotations against the NCBI nr database in different insect orders

Insect order	Salivary gland specific assembly	Gut specific assembly	Combined assembly
Hemiptera	43.16%	40.88%	40.63%
Blattaria	21.88%	21.35%	22.15%
Coleoptera	4.69%	4.13%	4.97%
Lepidoptera	5.69%	3.82%	3.01%
Hymenoptera	2.41%	2.41%	2.42%
Diptera	0.51%	0.62%	0.57%
Others	21.66%	26.78%	26.26%

**Fig. 2:**
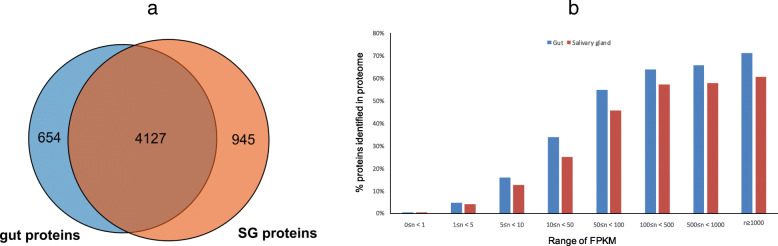
Summary of proteins mapped to proteomic peptides. Panel **a** Number of proteins identified in the SG, gut or both tissues. Panel **b** Correlations between transcript abundance and the probability of proteins mapped to proteomic peptides. Translated protein sequence sets derived from the salivary glands and gut were respectively used as targets for mapping of peptides that resulted from the proteomic analysis of the corresponding tissue. The proportion of proteins detected from the proteome increased with increasing FPKM values

To investigate the expression of the *E. onukii* genes at the protein level, we used the assembled transcripts as a database and mapped SG and gut protein peptide sequences resulting from proteomic sequencing to the protein sequences translated from the assembled contigs. The peptide mapping results are summarized in Additional file 1: Table S1. In total, 4457 unique transcripts in the SG and 3784 transcripts in the gut were mapped by the peptides derived from proteomic sequencing. The numbers of mapped proteins from the SG, gut and both tissues are shown in Fig. 2a. The majority of proteomic peptide mapped transcripts were identified in both the SG and gut, with 18.6% (945) and 13.7% (654) of transcripts predicted to be specifically expressed in the SG and gut, respectively. These results indicated that the SG and gut of *E. onukii* generally provide common functions in the digestive system. However, unique proteins identified in the SG and gut reflect that the two tissues also have different biological functions in *E. onukii*. In addition, certain numbers of transcripts identified from the proteomic profiles were associated with an increase in the FPKM (expected number of fragments per kilobase of transcript sequence per million base pairs sequenced) value of the transcripts (Fig. 2b), which was similar to previous observations in the investigation of stinkbug proteases [21, 26].

3) Identification of protease genes

We identified 930 unique contigs encoding putative proteases from SG and gut tissues by analyzing the BLAST annotation results. These proteases included aminopeptidase, carboxypeptidase, dipeptidase, aspartic protease, cathepsin B-like protease, cathepsin L-like protease and serine protease (trypsin-, chymotrypsin- and elastase-like protease). However, only 129 (14%) of the protease proteins were mapped by at least two unique peptides derived from proteomic sequencing profiles (Table 2), which included 26 aminopeptidases, 12 carboxypeptidases, 11 dipeptidases, 8 aspartic proteases, 14 cathepsin B-like proteases, 18 cathepsin L-like proteases and 40 serine proteases (Additional file 3). Fifty-two of the putative proteases mapped by proteomic profiles contained signal peptides that were potential digestive proteases. The other 77 proteases had either no signal peptides or the signal peptides could not be determined due to a lack of N-terminal sequences (Additional file 3). The protein sequences of the possible secreted proteases in the gut or SG of *E. onukii* are provided in Additional file 4.

4) Expression of protease transcripts

**Table 2:** Summary of identified digestive proteases

Category of protease	Nr annotated contig	Proteome identified	Percentage
Aminopeptidase	148	26	18%
Carboxypeptidase	89	12	13%
Dipeptidase	46	11	24%
Aspartic protease ^a^	39	8	21%
Cathepsin B-like	68	14	21%
Cathepsin L-like	197	18	10%
Serine protease ^b^	343	40	12%
Total	930	129	14%

^a^ Aspartic protease includes cathepsin D and aspartic protease

^b^ Serine protease includes trypsin, chymotrypsin and elastase

**Fig. 3:**
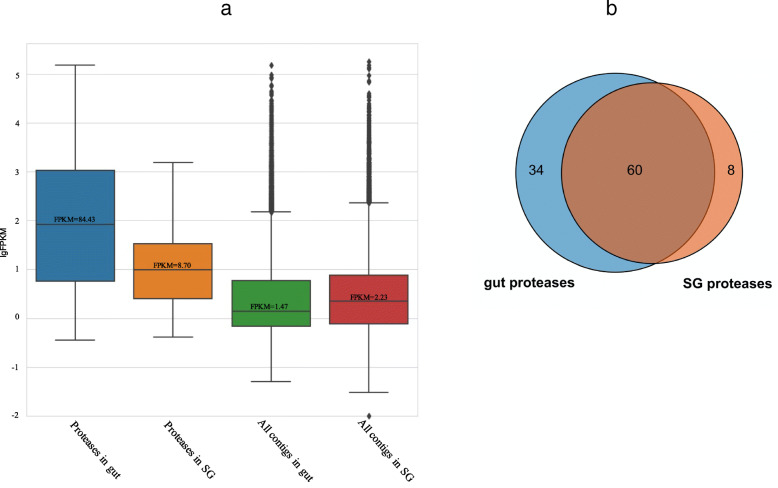
Transcriptomic abundance of proteases mapped to proteomic peptides. Panel **a** Median FPKM of predicted proteins in the SG and gut of *E. onukii*. Error bars indicate the standard deviation of the median FPKM of proteins in three biological replications. Panel **b** Number of proteases identified in the SG, gut or both tissues

To assess the overall expression of the transcripts in the SG and gut, the FPKM of each contig was estimated. The FPKM data were converted to log scale, and boxplots showing the medians and full range of FPKM variations are presented in Fig. 3a. In addition, boxplots using the FPKM values of transcripts encoding proteases are also shown in Fig. 3a. The majority of the transcripts had very low FPKM values, and the median FPKM was only 1.47 for the gut and 2.23 for the SG (Fig. 3a). On the other hand, proteases had much higher RNA expression levels. The median FPKM of proteases was 84.43 in the gut, which was 54-fold higher than the median FPKM calculated from the FPKM of all transcripts (Fig. 3a). Notably, 7 of the top 10 most abundant transcripts in the gut were proteases (Additional file 5). Transcripts of protease in the SG were lower than those in the gut of *E. onukii* but still higher than the average FPKM of the total SG contig set. The median FPKM of proteases in the SG was 8.70, which was approximately 4 times higher than that of total transcripts (Fig. 3a), although no transcripts of protease were among the top 100 most expressed proteins in the SG (Additional file 5).

The FPKM of each identified protease in the SG or gut is listed in Additional file 3 and presented by a heatmap (Additional file 2: Fig. S2). Of all the identified proteases, 107 had an FPKM≥1000 or 50 ≤ FPKM< 1000. The FPKM values of 49 proteases were higher than 1000 in the gut, while the FPKM values of only 10 proteases were above 1000 in the SG (Additional file 3). Differential analysis by DESeq2 showed that 85 out of 129 putative protease genes had over twice the expression level in the gut compared to the SG and only 13 putative protease genes had more than twice the expression level in the SG than in the gut (Additional file 3). Different types of proteases apparently differentially expressed in the SG or gut. Cathepsin-like proteases and serine proteases were the most abundant proteases among all proteases analyzed. In addition, the highly transcribed cathepsin B- and cathepsin L-like proteins in the gut also showed relatively higher FPKM values in the SG than the other cathepsin-like proteins (Additional file 3). Serine proteases were also abundant in the gut; 7 out of 40 potential serine proteases had FPKM values over 10,000. On the other hand, only two serine proteases (EMoSerineProtease-24 and -26) showed FPKM values over 1000 in the SG (Additional file 3). In the aminopeptidase group, EMoAminopeptidase-10 was the only aminopeptidase showing an FPKM over 1000 in the gut. Aminopeptidases were also highly transcribed in the SG of *E. onukii*. FPKMs of EMoAminopeptidase-1 and EMoAminopeptidase-3 in the SG were 710.05 and 755.95, respectively, which were 3.50 and 6.18 times higher than their expression levels in the gut, respectively (Additional file 3). Compared to other protease groups, carboxypeptidases and dipeptidases showed lower expression. EMoCarboxypeptidase-3 was the most abundant carboxypeptidase in the gut, and EMoCarboxypeptidase-5 and -6 were moderately transcribed (FPKM below 100). Conversely, EMoCarboxypeptidase-7, − 8 and − 9 were abundant in the SG but were less transcribed in the gut. In addition, the most abundant dipeptidase was EMoDipeptidase-2 (FPKM = 402.60) in the gut.

5) Tissue-specific distributions of protease proteins

The number of potential secreted proteases identified from the SG and gut is shown in Fig. 3b. The majority of the proteases (59%) were distributed in both tissues, with 8 and 34 proteases specific to the SG and gut, respectively. Among protease groups, the same number of aminopeptidases was found in the SG and gut, while more cathepsin-like proteases and serine proteases were found in the gut than in the SG (Additional file 1: Table S2). These results were consistent with the results of enzymatic activity tests, in which higher cathepsin and serine protease activities were observed in the gut; however, few differences in aminopeptidase activities were observed between the SG and gut. Various numbers of proteases identified in the SG and gut provided explanations of the enzymatic activities. Notably, EMoCathepsin L-16 and EMoSerineProtease-21 were highly expressed in the gut, with FPKMs of 44,267 and 3460, respectively (Additional file 3). However, from the proteomic profiles, these protease proteins were found only in the SG and not in the gut.

### Comparison of the top expressed proteases in *E. onukii* and five other hemipterans

The SG and gut of *E. onukii* expressed similar proteases (Table 3), which suggested that the SG and gut of *E. onukii* may play similar roles in food digestion. To determine whether other hemipteran insects have similar protease distributions, we analyzed available RNA-Seq data on proteases isolated from the SGs or guts of five hemipteran insects, including two rice planthoppers (*N. lugens* and *L. striatellus*), one rice leafhopper (*N. cincticeps*), one stink bug (*H. halys*) and one aphid (*A. pisum*). Information on the proteases in *N. lugens*, *L. striatellus*, *A. pisum*, *H. halys* and *N. cincticeps* is shown in Additional file 6. Due to the lack of genomic data for *N. cincticeps*, translated protein sequences of predicted proteases in *N. cincticeps* are shown in Additional file 4. The top ten proteases of each insect based on FPKM values were selected and are listed in Table 3. The results showed that aminopeptidase, carboxypeptidase and dipeptidase were among the most highly expressed proteases in the transcripts of the SG of *N. lugens*, *L. striatellus*, *A. pisum* and *N. cincticeps*. Compared with results for these four insects, cathepsin-like proteases and serine proteases were among the most highly expressed proteases in the SG of *E. onukii* and *H. halys* (including PSG and accessory SG, ASG) and only one carboxypeptidase was included in the top 10 most abundant digestive proteases in the SG of *E. onukii* and the PSG of *H. halys*. The most abundant transcripts of proteases were serine proteases (trypsin- and chymotrypsin-like) or cysteine proteases (cathepsin B- and L-like) in the gut of these hemipterans (RNA-Seq data of *N. cincticeps* gut is unavailable), including *E. onukii*. Cathepsin L-like protease is the most abundant protease family in the gut of *E. onukii* and *H. halys*.

**Table 3:** Top 10 digestive proteases abundant in the salivary gland and gut

*N. lugens*		*L. striatellus*		*A. pisum*		*H. halys*			*N. cincticeps*	*E. onukii*	
Salivary gland	Gut	Salivary gland	Gut	Salivary gland	Gut	Primary salivary gland	Accessory salivary gland	Gut	Salivary gland	Salivary gland	Gut
NlCathepsinB_4	NlSerineProtease_1	LsCarboxypeptidase_10	LsSerineProtease_1	ApAminopeptidase_14	ApCathepsinB_8	HhSerineProtease_25	HhSerineProtease_5	HhCathepsinL_8	NcAminopeptidase_9	EMoSerineProtease-24	EMoCathepsin L-15
NlCathepsinL_1	NlCathepsinB_3	LsCarboxypeptidase_7	LsCathepsinB_1	ApCathepsinL_1	ApAminopeptidase_3	HhSerineProtease_9	HhSerineProtease_29	HhCathepsinL_5	NcAminopeptidase_16	EMoCathepsin L-15	EMoCathepsin L-4
NlAminopeptidase_10	NlSerineProtease_2	LsCarboxypeptidase_3	LsSerineProtease_17	ApAminopeptidase_26	ApDipeptidase_2	HhSerineProtease_27	HhSerineProtease_23	HhCathepsinL_7	NcAminopeptidase_10	EMoSerineProtease-26	EMoSerineProtease-24
NlAminopeptidase_4	NlCathepsinB_5	LsCathepsinB_1	LsAsparticProtease_1	ApAminopeptidase_19	ApAminopeptidase_5	HhCarboxypeptidase_27	HhCathepsinL_1	HhCathepsinB_3	NcAsparticProtease_1	EMoCathepsin L-5	EMoCathepsin L-5
NlCathepsinL_3	NlSerineProtease_4	LsDipeptidase_3	LsSerineProtease_16	ApDipeptidase_2	ApCathepsinB_9	HhSerineProtease_26	HhCarboxypeptidase_16	HhCathepsinL_10	NcCathepsinB_1	EMoCathepsin L-4	SerineProtease-26
NlAsparticProtease_1	NlCathepsinB_1	LsAminopeptidase_14	LsSerineProtease_19	ApAminopeptidase_3	ApCathepsinB_1	HhSerineProtease_18	HhCathepsinL_21	HhCathepsinL_14	NcCathepsinL_5	EMoCathepsin L-3	EMoCathepsin L-3
NlCarboxypeptidase_1	NlSerineProtease_7	LsCarboxypeptidase_11	LsSerineProtease_18	ApCathepsinF_1	ApCathepsinB_6	HhSerineProtease_27	HhCathepsinB_1	HhCathepsinL_13	NcAsparticProtease_2	EMoCathepsin L-14	EMoCathepsin L-16
NlDipeptidase_1	NlAminopeptidase_3	LsAsparticProtease_2	LsCathepsinB_3	ApAminopeptidase_10	ApCathepsinB_11	HhSerineProtease_24	HhCarboxypeptidase_1	HhAminopeptidase_11	NcAminopeptidase_8	EMoCathepsin L-16	EMoSerineProtease-12
NlSerineProtease_1	NlSerineProtease_6	LsAminopeptidase_11	LsSerineProtease_15	ApCarboxypeptidase_1	ApCathepsinB_5	HhSerineProtease_4	HhAsparticProtease_2	HhCathepsinL_9	NcCathepsinL_3	EMoSerineProtease-12	EMoCathepsin B-6
NlAminopeptidase_11	NlAminopeptidase_2	LsAminopeptidase_5	LsSerineProtease_12	ApCathepsinK_1	ApSerineProtease_3	HhSerineProtease_28	HhSerineProtease_19	HhCathepsinL_4	NcAminopeptidase_14	EMoCarboxypeptidase-7	EMoCathepsin L-14

Detailed information of proteases in the table are shown in Additional files 3 and 6

### Phylogenetic analysis of potential digestive proteases isolated from *E. onukii* and other hemipteran insects

Phylogenetic trees were constructed for the aminopeptidases, cathepsin B- and L-like proteins and serine proteases isolated from SGs and guts of six hemipteran insects (*E. onukii*, *A. pisum, H. halys, N. cincticeps, N. lugens* and *L. striatellus*). In addition, phylogenetic analyses of the proteases from hemipteran insects and other insect groups were also performed.

1. Aminopeptidase

**Fig. 4:**
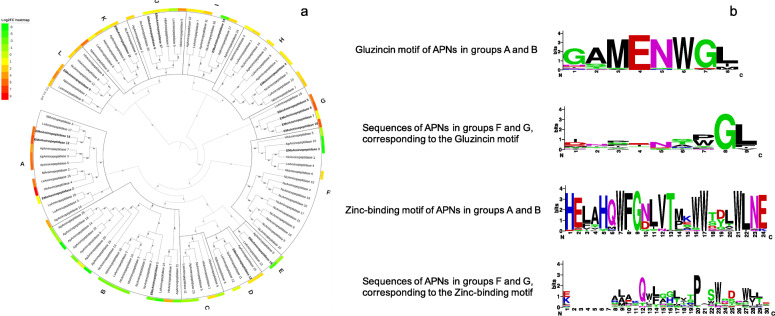
Phylogenetic tree containing aminopeptidases from six hemipteran species. Protein sequences of aminopeptidase-like proteins from *Nilaparvata lugens* (Nl), *Laodelphax striatellus* (Ls), *Nephotettix cincticeps* (Nc), *Acyrthosiphum pisim* (Ap), *Halyomorpha halys* (Hh) and *Empoasca* (Matsumurasca) *onukii* (EMo) were aligned and used for the phylogenetic analysis through the maximum likelihood strategy (Panel **a**). Information regarding the proteins is shown in Files S3 and S6. Proteins from *E. onukii* are indicated in bold. Proteins in the phylogenetic tree mentioned in the paper are arrowed. Groups A, B, F and G: proteins annotated by aminopeptidase N; Group C: glutamyl aminopeptidase; Group D: puromycin sensitive aminopeptidase; Group E: endoplasmic reticulum aminopeptidase; Group H: methionine aminopeptidase; Group I: xaa-Pro aminopeptidase; Group J: aminopeptidase NPEPL1; Group K: cytosol aminopeptidase; Group L: aminopeptidase W07G4.4e. Relative expression level is presented by log_2_FC (gut vs SG) and shown by a colored circle outside the tree, from green (log_2_FC = −6) to red (log_2_FC = 9). Proteins with no log_2_FC of FPKM are left blank. The sequence logos (logs?) of the gluzincin motif and zinc-binding motif from aminopeptidase N-like proteins in groups A, B, F and G are shown in (panel **b**)

Many types of aminopeptidases have been identified [28]. Nine different aminopeptidase groups were identified in the transcriptome of the SG and gut of *A. pisum, H. halys, N. cincticeps*, *N. lugens*, *L. striatellus* and *E. onukii*. The phylogenetic analysis grouped the aminopeptidases into 12 clades (Fig. 4a). Aminopeptidase N (APN) was the largest group and distributed in four clades (A, B, F and G). The remaining aminopeptidases were divided into eight clades representing a variety of aminopeptidase groups (Fig. 4a). Insect aminopeptidase N normally contains a gluzincin motif (GXMEN) and a zinc-binding motif (HEXXHX^18^E) [29]. We noticed that these two motifs were only observed in the APNs in groups A and B but not in groups F and G. Groups F and G were mainly composed of deduced aminopeptidases of *E. onukii* and *N. cincticeps*, respectively (Fig. 4b). In addition, the APNs of group A generally had higher transcriptional levels in the gut than in the SG of the five hemipteran insects. In contrast, group B APNs showed higher expression levels in the SG than in the gut (Fig. 4a). The differentially expressed APNs of groups A and B found in the SG and gut were uniquely clustered when the APNs of more insect orders were introduced for phylogenetic tree construction (Additional file 2: Fig. S3).

2) Cathepsin B- and L-like proteins

**Fig. 5:**
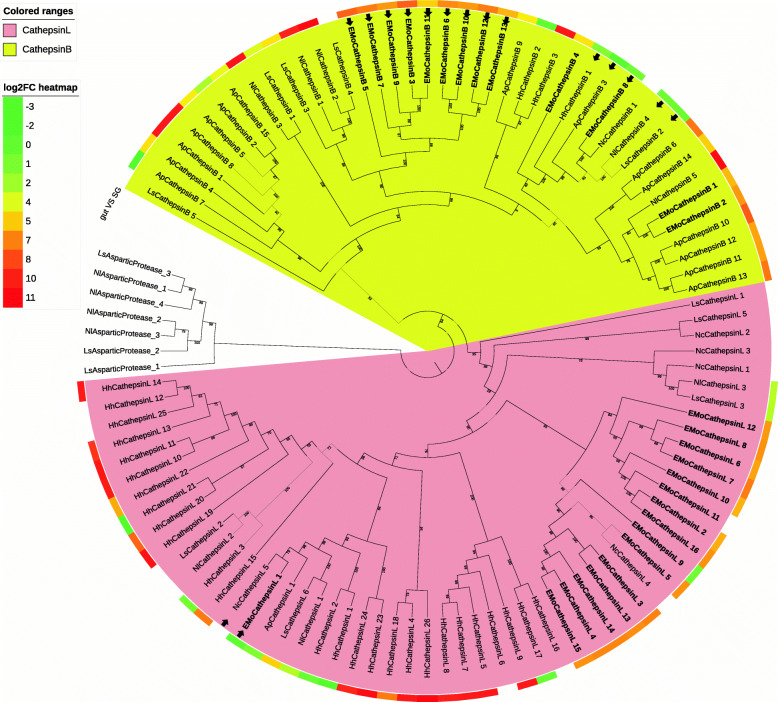
Phylogenetic tree containing cathepsin B- and cathepsin L-like proteins from six hemipteran species. Protein sequences of cathepsin B- and cathepsin L-like proteins from *N. lugens* (Nl), *L. striatellus* (Ls), *N. cincticeps* (Nc), *A. pisim* (Ap), *H. halys* (Hh) and *E. onukii* (EMo) were aligned and used for the phylogenetic analysis through the maximum likelihood strategy. Cathepsin D-like proteins of *N. lugens* and *L. striatellus* were clustered as the outgroup. Information regarding the proteins is shown in Files S3 and S6. Proteins from *E. onukii* are indicated in bold. Proteins in the phylogenetic tree mentioned in the paper are arrowed. The clade highlighted by pink and yellow contains cathepsin B- and cathepsin L-like proteins, respectively. Relative expression level is presented by log_2_FC (gut vs SG) and shown by a colored circle outside the tree, from green (log_2_FC = −3) to red (log_2_FC = 11). Proteins with no log_2_FC of FPKM are left blank

Cathepsin B- and L-like proteins identified from the SG and gut of *E. onukii* and five other hemipterans were clearly grouped into two distinct clusters in the phylogenetic tree: a cathepsin B group and a cathepsin L group (Fig. 5). In the group containing cathepsin L-like proteins, the cathepsin L-like proteases of *E. onukii* clustered together in the same clade except for EMoCathepsin L-1, which is closely related to NcCathepsin L-5 (Fig. 5). Cathepsin B-like proteases, which showed higher transcriptional levels in the gut of *E. onukii* (EMoCathepsin B -3, − 5, − 6, − 7, − 9, − 10, − 11, − 12 and − 13), were clustered together. On the other hand, EMoCathepsin B-8, the only cathepsin B-like protein of *E. onukii* showing a higher expression level in the SG, was grouped with four other SG-enriched cathepsin B-like proteases: HhCathepsin B-1, ApCathepsin B-3, LsCathepsin B-2, and NlCathepsin B-4 (Fig. 5).

3) Serine protease

**Fig. 6:**
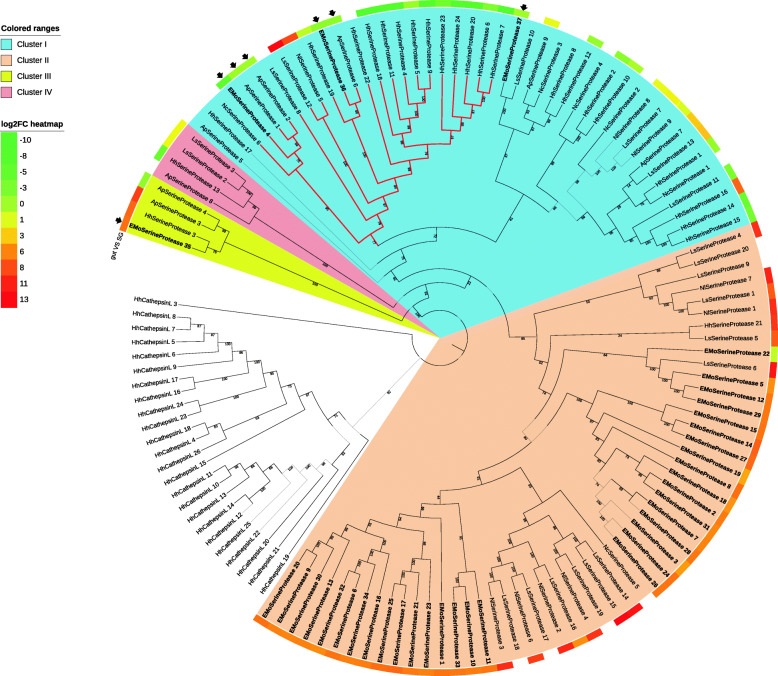
Phylogenetic tree containing serine protease-like proteins from six hemipteran species. Protein sequences annotated by trypsin, chymotrypsin and elastase from *N. lugens* (Nl), *L. striatellus* (Ls), *N. cincticeps* (Nc), *A. pisim* (Ap), *H. halys* (Hh) and *E. onukii* (EMo) were aligned and used for the phylogenetic analysis through the maximum likelihood strategy. Information regarding the proteins is shown in Files S3 and S6. Proteins from *E. onukii* are indicated in bold. Proteins in the phylogenetic tree mentioned in the paper are arrowed. Clades highlighted by blue and orange indicate two clusters of serine protease. The branch highlighted by red indicates a cluster of serine protease showing relatively higher FPKMs in the salivary gland than in the gut. Relative expression level is presented by log_2_FC (gut vs SG) and shown by a colored circle outside the tree, from green (log_2_FC = − 10) to red (log_2_FC = 13). Proteins with no log_2_FC of FPKM are left blank

The serine proteases (trypsin-like, chymotrypsin-like, elastase-like and other serine proteases) of *E. onukii* and five other hemipteran insects were mainly divided into two major clusters in the phylogenetic tree (Fig. 6). Most of the cluster I serine proteases showed low to moderate transcriptional abundance in the gut, while most of the cluster II serine proteases showed higher expression in the gut. Significantly, a branch of serine proteases in cluster I, including NcSerineProtease-6, EMoSerineProtease-4, − 36, ApSerineProtease-1, − 2, − 6, and multiple other serine proteases, which were highly transcribed in the SG of stink bugs [21, 26], were grouped together (the clade colored red in Fig. 6). The putative serine proteases from *E. onukii* were mostly grouped into cluster II with the exception of EMoSerineProteases-4, − 35, − 36 and − 37 (Fig. 6). In addition to the two major clusters, EMoSerineProtease-35 and seven other serine proteases from *A. pisum, H. halys,* and *L. striatellus* were clustered into two small clades independent from other serine proteases (clusters III and IV). To understand whether the cluster II serine proteases were distinct from the serine proteases abundant in the SG, venom serine proteases from various insect orders were appended for phylogenetic analysis. Interestingly, when the venom serine proteases of multiple insect orders were included in the phylogenetic analysis, the serine proteases in cluster II, which were highly expressed in the gut of hemipterans, were again grouped into a distinct clade and located at the root of the tree (Additional file 2: Fig. S4).

## Discussion

The primary goal of this study was to identify digestive proteases in *E. onukii* by analyzing transcriptomic and proteomic profiles derived from the SG and gut of *E. onukii*. We discovered at least 129 protease sequences, 52 of which contained signal peptides that were putative digestive proteases. These proteases include aminopeptidase, carboxypeptidase, dipeptidase, aspartic protease, cysteine protease (cathepsin B- and L-like), and serine protease (trypsin-, chymotrypsin- and elastase-like protease). Furthermore, cysteine proteases and serine proteases showed significantly higher activity in the gut than in the SG, while almost equal enzymatic activity of aminopeptidase was determined in both tissues (Fig. 1). Serine proteases (trypsin- and chymotrypsin-like proteases) and cysteine proteases (cathepsin B- and cathepsin L-like) were identified as the major digestive enzymes in the midguts of phloem sap-sucking hemipterans, although phloem sap is nutritionally inadequate [21–23, 26, 30, 31]. Our results are consistent with previous investigations of digestive proteases of hemipteran insects. It is interesting to observe that both cysteine and serine proteases accumulate in the guts of hemipteran insects, yet these two groups of proteases require a different pH for their activity. The optimum environment for cysteine proteases is acidic (pH < 7.0), while neutral and alkaline pH values are conducive to serine proteases [31, 32]. One possible explanation for the existence of both cysteine and serine proteases in *E. onukii* is that gut pH varies through their digestive tracts. *Pondus hydrogenii* variation in the midgut of *Aphis gossypii* was observed alongside changes from acidic (stomach) to lower alkaline (central and posterior midgut) [33].

Insect digestive proteases are produced mainly by midgut epithelial cells and secreted into the lumen, where the food bolus passes through [34, 35]. However, transcription or translation of digestive proteins could be regulated by insect feeding behaviors, developmental stages, and food sources. For instance, enzymatic activities in the SG and midgut of the mirid bug *Apolygus luncorum* are regulated by sex, age and food resources (plant or animal sources) [22]. In addition, symbiotic bacteria can also regulate digestive protease expression [30]. Differential expression of various proteases in the SG and gut is also observed in pentatomid stink bugs, *N. viridula* [26] and *H. halys* [21]. Compared to these two species, *E. onukii* appears to have a very different regulation of protease expression in its digestive system. In general, more proteases were identified in the gut from both transcriptomic and proteomic data in *E. onukii*. Of the 129 protease proteins mapped by the peptide profiles, only 16 proteases were unique in the SG, and 2 (EMoCathepsin L-16 and EMoSerine protease-21) of them had a much higher transcription level (log_2−_fold changes, log_2_FC > 6.5) in the gut (Additional file 3). These results suggested that some proteases, especially cathepsin-like proteases and serine proteases, might be transferred from the gut to the SG. Two highly expressed cathepsin-like proteases in the gut of *H. halys* were detected in saliva [21]. These results imply that cathepsin-like proteases in *H. halys* could possibly be delivered from the gut to saliva. An earlier investigation of the aphid *Tuberaphis styraci* suggested that 1st instar aphid soldiers could inject midgut-expressed cathepsin B-like proteases through their stylets into enemies [36]. Our observation seems to support the proposition that hemipteran insects may utilize midgut-expressed proteases for extraoral digestion, although the actual mechanism remains to be discovered. Studies of tea leaf protein composition show that 15% of the dry weight of tea leaves is proteins [37]. Therefore, a high abundance of digestive enzymes in the gut of *E. onukii* may be functional in the digestion of ingested proteins from the mesophyll and stem parenchyma cells of tea plants.

Recently, transcriptomic and proteomic analyses identified various proteases in the saliva of *H. halys*. The vast majority of the proteases found in saliva are serine proteases that are highly expressed in the PSG of *H. halys* but also include cathepsin-like proteases and peptidases [21]. Significantly, almost all the proteases recovered from saliva are proteases with the highest transcript level, suggesting that proteases with a higher transcriptional level are more likely to be functional in food digestion. Hence, we analyzed the transcriptomes of five hemipteran insects, including two planthoppers (*N. lugens* and *L. striatellus*), one leafhopper (*N. cincticeps*), one aphid (*A. pisum*) and one stink bug (*H. halys*). The top ten most highly expressed proteases in the SG and gut of *E. onukii* were compared to the findings in those five hemipterans (Table 3). As expected, the top 10 proteases in the PSG of *H. halys* included nine serine proteases and one carboxypeptidase that have been previously found in the saliva of *H. halys* [21]. These results further prove that proteases with a high transcriptional level in digestion-related organs are likely to be involved in food digestion. It is clear that the top 10 most highly expressed protease transcripts (mainly cathepsin-like and serine proteases) in the SG and gut of *E. onukii* were very similar, indicating that both the SG and gut of *E. onukii* play important roles in food digestion. The SGs and guts of the three rice feeders (*N. lungens*, *L. striatellus* and *N. cincticeps*) presented similar protease compositions (Table 3). In the guts of the three rice feeders and one aphid, cathepsin-like proteases or serine proteases were the most abundant proteases. In addition to cathepsin-like and serine proteases, aminopeptidases were also included in the top expressed proteases in the SGs of these four hemipterans. It is not clear whether the aminopeptidases were released into the saliva. No aminopeptidase was identified in the saliva of *H. halys*, although low to moderate expression of aminopeptidases was found in the SG of *H. halys* and *N. viridula* [21]. In the present study, no aminopeptidase was included in the top ten lists of the PSG and ASG of *H. halys* (Table 3). It is believed that *E. onukii* is a typical cell-rupture feeder [1], and *H. halys* was reported to have both cell-rupture and sheath forming feeding behaviors [38]; however, the other hemipterans investigated in this report are sheath forming feeders [12, 39–42]. Cell-rupture feeders feed intracellularly on the mesophyll and parenchyma cells by rapidly moving their stylets and continually secreting saliva to digest plant tissues in vitro, followed by the sucking of this processed soup through the stylets [14]. Hence, ingested plant juices containing rich protein components require further processing in the alimentary tract. This predicted feeding behavior might explain why the SG and gut of *E. onukii* contain similar digestive proteases. On the other hand, sheath-forming hemipterans generally suck sap from vascular tissues (phloem and xylem) [14, 43]. Hence, the nutritional components of sap ingested from vascular tissues are significantly different from the juices of broken mesophyll and parenchymal cells in nonvascular tissues. The SGs of the three rice feeders (*N. lungens*, *L. striatellus* and *N. cincticeps*) presented similar protease compositions (Table 3). Consequently, different feeding behaviors and food sources may play important roles impacting protease compositions in the SGs of hemipterans.

Phylogenetic analysis of aminopeptidases from six hemipteran insects showed that the APNs of hemipterans, which contain a gluzincin motif and a zinc-binding motif, were divided into two distinct clades. One clade (A) had a higher transcript level in the gut, while the APNs of the other clade (B) were more abundant in the SG (Fig. 4). In the phylogenetic tree containing APNs from multiple insect orders, the hemipteran APNs in clades A and B of the phylogenetic tree in Fig. 4 were themselves separated into two clades (Additional file 2: Fig. S3). Notably, the APNs of other insect families were also divided into two clusters (Additional file 2: Fig. S3). Hence, the APNs in insect digestive tissues may be of two types, one mainly expressed in the SG and the other highly expressed in the gut. Further study is needed to clarify the current observations.

Similar to the clusters of hemipteran APNs, the majority of serine proteases from the six hemipteran insect species were divided into two distinct clusters at the root of the phylogenetic tree (Fig. 6). Based on the transcriptomic level, some serine proteases in cluster I (the red clade) are likely to be the venom proteases that were highly transcribed in the SG, while the majority of cluster II serine proteases were abundant in the gut. Interestingly, with the addition of venom serine proteases from other insect orders, the cluster II hemipteran serine proteases formed a unique branch distinct from all other venom serine proteases from multiple orders of insects, including Hemiptera (Additional file 2: Fig. S4). These results indicate that serine proteases were divided into a gut-abundant group and a venom group prior to the emergence of Hemiptera.

## Conclusions

In summary, we found that both the SG and gut of *E. onukii* express similar groups of proteases and most of the proteases were highly transcribed in the gut. We also compared the most highly expressed proteases in the SG and gut of *E. onukii* with the results for five other hemipteran insects and found differences in the protease distributions in the five analyzed hemipterans relative to *E. onukii*. The variation in the proteases expressed in the SG and gut could be associated with the insects’ feeding behaviors and food sources. The phylogenetic analysis suggests that proteases highly expressed in the SG or gut evolved in two distinct directions. Tea cultivars resistant to infestation by *E. onukii* have been previously reported [11, 44]. Protease inhibitors are potential biocontrol agents for insect pests [45–47]. The information presented here enriches our understanding of the digestive systems of *E. onukii* and could provide targets for the development of protease inhibitors and other biocontrol agents to manage this important tea pest. This work could also provide a knowledge base for the exploration of interaction mechanisms between tea plants and *E. onukii.*

## Methods

### Insects, tissue collections and sample preparations

**Fig. 7:**
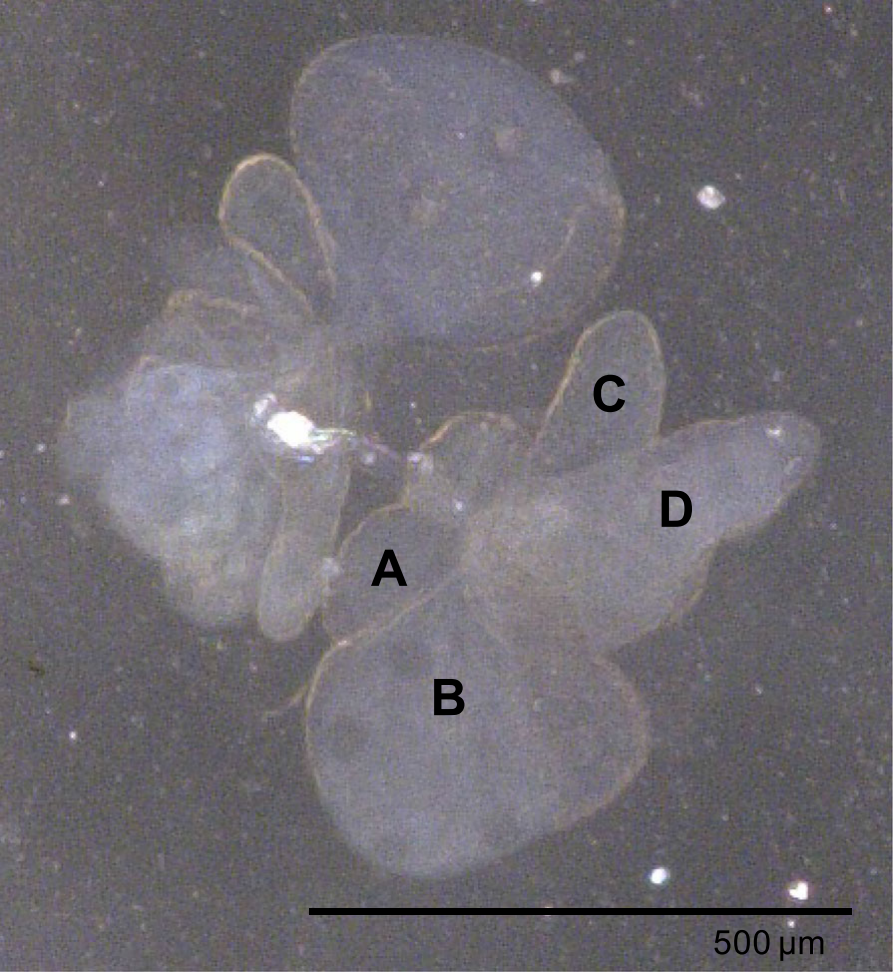
Excised salivary glands of *Empoasca* (Matsumurasca) *onukii* nymphs. Salivary glands were dissected and imaged by digital microscope VHX-2000C (KEYENCE, Japan). **a**-**d** indicate four lobes of one side of the salivary gland

The *E. onukii strain* used in this research was originally collected from a tea field located at Fujian Agriculture and Forestry University, Fuzhou, Fujian Province, China, and has been maintained in laboratory conditions for approximately 2 years. The *E. onukii* colony was raised on tea shoots, bred in water and kept at 28 °C with a photoperiod of 14:10 (light:dark) h in an insectary. The 3rd-instar nymphs of *E. onukii* were collected from water-planted fresh tea shoots and used for isolation of guts and salivary glands. To dissect the gut and salivary gland, insects were immobilized on ice for several minutes before they were dissected. SG isolation was performed under a stereomicroscope (VHX-5000, KEYENCE, Japan). The morphology of the SG is illustrated in Fig. 7. Morphological images of SG tissues were obtained via digital microscopy (VHX-2000C, KEYENCE, Japan). The methods used for gut dissections have been previously described [27].

To determine the enzyme activities, six hundred salivary glands or guts were homogenized in 600 μL PBS (137 mM NaCl, 2.68 mM KCl, 8.1 mM Na_2_HPO_4_, 1.47 mM KH_2_PO_4_, pH 7.4) in a 1.5 mL microcentrifuge tube. Preparation of the homogenized samples was replicated three times, and the protein concentrations were normalized by the results of the Bradford method [48]. For the RNA extractions, approximately 1500 SGs and 1000 guts were isolated, and the tissues were quickly washed in a diethylpyrocarbonate (DEPC)-treated PBS solution after dissection. The tissues were kept in RNAhold (TransGen Biotech, China). For proteomic sequencing, 2500 SGs or 1500 guts were quickly washed and resuspended in PBS. The tissues were then immediately frozen with liquid nitrogen and kept at − 80 °C. Tissue collections for RNA sequencing and proteomic analysis were repeated three times.

### Activity assays of digestive proteases

For the enzyme activity assays, collected SG or gut tissues were homogenized by a mortar homogenizer. Leucine aminopeptidase activity was measured using leucine p-nitroanilide (LpNA) (Sigma, US) as a chromogenic substrate [49]. Tissue homogenates were diluted to 100 μg/mL by 0.1 M Tris-HCl (pH 8.6). Eighteen micrograms of each sample was mixed with 1 mM prewarmed (30 °C) substrate buffer (0.1 M Tris-HCl, 1 mM LpNA, pH 8.6). The enzymatic reaction was monitored for increases in optical absorbance at 405 nm and 30 °C in a microplate reader (SPARK 10 M, Tecan) for 10 min (observations were carried out every 10 s, and 60 cycles were conducted for each reaction). Trypsin activity was determined by mixing 5 μg protein in 3 mL of 1 mM Na-benzoyl-L-arginine p-nitroanilide (BApNA, Sigma, US) in 50 mM Tris-HCl (pH 8.0). The enzymatic reactions at 28 °C were monitored in a UV-VIS spectrophotometer (TU-I950, PERSEE) by recording the optical absorbance at 405 nm for 40 min (observations were carried out every 30 s, and 80 cycles were conducted for each reaction). The activities of cathepsin L- and B-like protease were assayed using carbobenzyloxy-Phe-Arg-(7-amino-4-methyl-coumarin) (Z-FR-AMC, Santa Cruz Biotechnology, US) as a fluorescent substrate. Briefly, 5 μg of the protein was suspended in activation buffer (10 mM Tris-HCl, pH 7.5) at a final volume of 60 μL, including Z-FR-AMC (0.1 mM). The enzymatic reactions were monitored at 30 °C in a microplate reader (SPARK 10 M, Tecan) with an optical absorbance increase at an excitation wavelength of 380 nm and an emission wavelength of 460 nm for 40 min (observations were carried out every 30 s, and 80 cycles were conducted for each reaction). One unit of enzyme (U) was defined as the amount that hydrolyzes 1 μmol of substrate per minute. Mean enzyme activities were calculated from three readings for one replication. Chymotrypsin activity assays were carried out using a chymotrypsin activity assay kit (A080–3-1, Nanjing Jiancheng Bioengineering Institute, China) according to the manufacturer’s instructions. All enzymatic reactions were replicated at least three times for the statistical analysis using Student’s *t*-test in Prism (version 8.2.0).

### RNA extraction, library preparation, Illumina sequencing and de novo assembly

Collected tissues were homogenized moderately with a hand homogenizer. Total RNA was extracted using an HP Total RNA Kit (Omega, USA) according to the manufacturer’s protocol. The quantity of extracted RNA was confirmed with a NanoDrop (Bio-Rad, USA), and the quality of RNA was verified by Agilent 2100 (Agilent, Germany) and electrophoresis gel analysis. Approximately 1.5 μg of total RNA extracted from each sample was used to generate sequencing libraries. Sequencing libraries were constructed using the NEBNext® Ultra™ RNA Library Prep Kit for Illumina® (NEB, USA) following the manufacturer’s recommendations. The qualified cDNA library was clustered through the Illumina cBot system and sequenced on an Illumina HiSeq 2500 platform to generate 150 nt paired-ended reads (Novogene Bioinformatics Technology Co., Ltd., Beijing, China). The original image data were processed with Illumina GA Pipeline v1.3 to clean reads, followed by the removal of adapter sequences, empty reads and low-quality reads. The clean reads obtained from SG or gut samples were uploaded to the NCBI SRA database (BioProject ID: PRJNA606974) and assembled by Trinity Assembly (version 2.8.5) [50] with the default parameter settings, and all reads pooled together were also assembled. Tissue-specific RNA sequencing reads from the SGs or guts of *Nilaparvata lugens* (SRR5149721 and SRR8189329), *Laodelphax striatellus* (SRR1617628 and SRR1617623), *Nephotettix cincticeps* (SRR018462), *Acyrthosiphum pisum* (SRR7037541 and SRR7037537) and *Halyomorpha halys* (SRX6717290, SRX6717291, SRX6717292) were downloaded from the NCBI Sequence Read Archive (SRA) database and separately assembled by Trinity as described above. To assess the completeness of the assembled data, the assembled transcripts were analyzed using arthropod gene sets in the BUSCO database as a reference by the BUSCO program [51].

### Transcriptome analysis

Functional annotation of the de novo assembled contigs was conducted by a BLASTx search against the NCBI nr (nonredundant) and Swiss-Prot databases with an E-value cutoff of 10^− 5^. The protein sequences of the contigs with positive hits were translated for further analysis. Potential coding sequences (CDSs) of the contigs without hits by BLAST annotation were predicted by ESTScan (version 3.0.3). Relative expression of the transcripts in the pooled assembled *E. onukii* datasets was estimated using RSEM software (version 1.3.1) [52]. Read mapping and FPKM values were performed by Bowtie. The FPKM of protease genes was divided by that of the reference gene into either the SG or gut for normalization. Reference genes from each insect species were selected according to previous studies [53–56]. Information on selected reference genes is shown in Additional file 6. The relative expression of protease genes in the SG and gut of *N. lugens*, *L. striatellus*, *A. pisum*, and *H. halys* is presented by calculating the log_2_FC of the FPKM values (gut vs SG). Median FPKM values were calculated and plotted by boxplot functions using a Python script. Heatmaps of the FPKM values were generated using TBtools software (version 0.6652) [57].

### Extraction of total protein and LC-MS/MS analysis

Tissue samples were thoroughly milled in liquid nitrogen by a mortar homogenizer. The milled powders were mixed with lysis buffer (50 mM Tris-HCl, pH 8.0, 8 M urea and 0.2% SDS) and incubated with ultrasonication on ice for 5 min, followed by centrifugation at 12,000 g for 15 min at 4 °C. Samples were mixed with 2 mM dithiothreitol and incubated at 56 °C for 1 h, followed by the addition of sufficient iodoacetic acid and incubation for 1 h at room temperature in darkness. The samples were then mixed with 4 volumes of cold acetone, vortexed and placed at − 20 °C overnight, followed by centrifugation at 12,000 g for 15 min at 4 °C. The supernatants were discarded, and the pellets were washed twice with cold acetone. The washed pellets were dissolved in dissolution buffer (0.1 M triethylammonium bicarbonate, pH 8.5, 8 M urea), and the protein concentration was determined by the Bradford assay. Protein samples were sent to Novogene Co., Ltd. (China) for further preparation followed by liquid chromatography (LC)-electrospray ionization (ESI) tandem mass spectrometry (MS/MS) analysis by Novogene Co., Ltd. (China).

### Identification of protease proteins from transcriptomic and proteomic data

Putative protease proteins were identified by searching for the positive hits of proteases from BLAST results. Protein sequences from three individual assemblies for the RNA-seq data (SG, gut and combined reads) that hit the same accession numbers were manually checked to determine whether they were derived from the same genes. Therefore, we only present each identified protease protein by a given ID name instead of the transcript IDs (Additional file 3). The assembled transcripts annotated as aminopeptidase, aspartic protease (cathepsin D), carboxypeptidase, dipeptidase, cathepsin B, cathepsin L, trypsin, chymotrypsin, elastase, cysteine protease and serine protease were identified as putative proteases. The putative protease fragments (> 100 aa) obtained from contig sets of the gut, SG and pooled reads were further examined by BLASTp to sort for duplicate protease sequences. Sequences that hit the same accession numbers were manually checked by alignment of these sequences using MEGAX (version 10.1.7) built-in MUSCLE and ClustalW programs to determine whether they were derived from the same genes. A similar process was also used to identify protease proteins from the transcriptomes of *N. lugens,* *L. striatellus, N. cincticeps* and *A. pisum* species. Signal peptidases of 5′ end complete proteins were predicted by the SignalP-5.0 server [58].

To identify protease proteins from the proteomic data, putative amino acid sequences of proteins encoded by the contigs assembled from the gut, SG and pooled reads were translated using TransDecoder (v5.5.0) [59]. The protein sequences were mapped to the peptide libraries that resulted from LC-MS/MS analysis using Discoverer 2.2 software (Thermo Fisher Scientific, Bremen, Germany). A protein sequence mapped by at least two different peptides was selected. Selected proteins predicted to contain a signal peptide at the N-terminus were determined as putative digestive proteases.

### Phylogenetic analysis

Aminopeptidase, cysteine proteases (cathepsin B- and cathepsin L-like protease) and serine proteases (trypsin-, chymotrypsin- and elastase-like protease) identified in *A. pisum, N. lugens, L. striatellus, N. cincticeps, H. halys* [21] and *E. onukii* were included in the phylogenetic analysis. Information about the proteins used in the phylogenetic analysis is shown in Additional files 3 and 6. For the phylogenetic analysis of aminopeptidase- or cathepsin-like proteins from multiple orders of insects, reference proteins were randomly selected from the BLASTp results using EMoAminopeptidases and EMoCathepsins as queries against the NCBI nr database, respectively. For the phylogenetic analysis of serine proteases from multiple insect orders, reference proteins were randomly selected from previously reported insect venom serine proteases [21]. Plant aminopeptidase N-like proteins, hemipteran aspartic proteases and cathepsin-like proteases of *H. halys* were included in the phylogenetic tree of aminopeptidase N, cathepsin-like protease and serine protease to form an outgroup. Protein sequences of each protease group were aligned in batches with MAFFT [60]. Aligned sequences were then assessed via phylogenetic analysis using the maximum likelihood (ML) method. ModelFinder [61] implemented in IQ-TREE [62] was used to choose the best partitioning scheme and models. ML analysis was performed using IQ-TREE with 10,000 ultrafast bootstraps [63]. Constructed trees were uploaded to the Interactive Tree of Life (http://itol.embl.de) for visualization and annotations.

## Supplementary Information

**Additional file 1.** Additional tables. This file contains 2 additional tables

**Additional file 2.** Additional figures. This file contains 4 additional figures

**Additional file 3 **Putative proteases in *E. onukii*. This file lists information of predicted proteases identified from salivary glands and guts of *E. onukii*

**Additional file 4 **Protein sequence of putative proteases in *E. onukii* and *N. cincticeps*. This file lists protein sequences of putative proteases in *E. onukii* and *N. cincticeps*

**Additional file 5 **The top 100 abundant proteins in the SG or gut of *E. onukii*. This file lists information on the top 100 abundant proteins in the SG or gut of *E. onukii*

**Additional file 6 **Information on insect protease proteins included in the analysis. This file lists information on protease proteins included in the analysis from other insect species besides *E. onukii*

## Data Availability

All raw sequences of *E. onukii* were deposited in the National Center for Biotechnology Information (NCBI) Sequence Read Archive (BioProject: PRJNA606974). Raw sequencing reads from SGs or guts of *Nilaparvata lugens* (SRR5149721 and SRR8189329), *Laodelphax striatellus* (SRR1617628 and SRR1617623), *Nephotettix cincticeps* (SRR018462), *Acyrthosiphum pisum* (SRR7037541 and SRR7037537) and *Halyomorpha halys* (SRX6717290, SRX6717291, SRX6717292) were downloaded from the NCBI Sequence Read Archive database. Accession numbers corresponding to some of the datasets analyzed in this study can also be found in Additional file 6 and are stored in the NCBI database.

## Abbreviations

SG: Salivary gland
PSG: Principle salivary gland
FPKM: Reads per kilobase per million mapped reads
SRA: Sequence read archives
PBS: Phosphatic buffer solution
RNA: Ribonucleic acid
cDNA: Complementary DNA
NCBI: National Center for Biotechnology Information
Log_2_FC: Log_2_ fold changes
CDS: Sequence coding for amino acids in protein
DEPC: Diethylpyrocarbonate
LpNA: leucine p-nitroanilide
BApNA: Na-benzoyl-L-arginine p-nitroanilide
Z-FR-AMC: Carbobenzyloxy-Phe-Arg-(7-amino-4-methyl-coumarin)
ANOVA: One-way analysis of variance
LC-MS/MS: Liquid chromatography - electrospray ionization tandem mass spectrometry
EMo: *Empoasca onukii*
Nl: *Nilaparvata lugens*
Ls: *Laodelphax striatellus*
Nc: *Nephotettix cincticeps*
Ap: *Acyrthosiphum pisum*
Hh: *Halyomorpha halys*

## References

[1] Jin S, Chen ZM, Backus EA, Sun XL, Xiao B (2012). Characterization of EPG waveforms for the tea green leafhopper, Empoasca vitis Göthe (Hemiptera: Cicadellidae), on tea plants and their correlation with stylet activities. J Insect Physiol.

[2] Peng P, Tang M, Hou Y-j, Lin Q, Huang S-j, Deng M, Hu X, Zhang Y (2010). Study on the Effect and Characters of Yellow Sticky Trap Sticking Aleurocanthus spiniferus and Empoasca vitis Gothe in Tea Garden. Southwest China J Agric Sci.

[3] Qin D, Zhang L, Xiao Q, Dietrich C, Matsumura M (2015). Clarification of the identity of the tea green leafhopper based on morphological comparison between Chinese and Japanese specimens. PLoS One.

[4] Zhang Z, Luo Z, Gao Y, Bian L, Sun X, Chen Z (2014). Volatiles from non-host aromatic plants repel tea green leafhopper Empoasca vitis. Entomologia Exp Appl.

[5] Wei Q, Yu H-Y, Niu C-D, Yao R, Wu S-F, Chen Z, Gao C-F (2015). Comparison of insecticide susceptibilities of Empoasca vitis (Hemiptera: Cicadellidae) from three Main tea-growing regions in China. J Econ Entomol.

[6] Guo H (2011). Research progress of tea tree major pest, Empoasca vitis. Jiangsu Agric Sci.

[7] Wei Q, Mu X-C, Yu H-Y, Niu C-D, Wang L-X, Zheng C, Chen Z, Gao C-F (2017). Susceptibility of Empoasca vitis (Hemiptera: Cicadellidae) populations from the main tea-growing regions of China to thirteen insecticides. Crop Prot.

[8] Bian L, Sun XL, Luo ZX, Zhang ZQ, Chen ZM (2014). Design and selection of trap color for capture of the tea leafhopper, E mpoasca vitis, by orthogonal optimization. Entomologia Exp Appl.

[9] Hazarika LK, Bhuyan M, Hazarika BN (2009). Insect pests of tea and their management. Annu Rev Entomol.

[10] Ye G-Y, Xiao Q, Chen M, Chen X-x, Yuan Z-j, Stanley DW, Hu C (2014). Tea: biological control of insect and mite pests in China. Biol Control.

[11] Yorozuya H (2017). Analysis of tea plant resistance to tea green leafhopper, E mpoasca onukii, by detecting stylet-probing behavior with DC electropenetrography. Entomologia Exp Appl.

[12] Sharma A, Khan A, Subrahmanyam S, Raman A, Taylor G, Fletcher M (2014). Salivary proteins of plant-feeding hemipteroids–implication in phytophagy. Bull Entomol Res.

[13] Miles PW. The saliva of Hemiptera. In: Advances in insect physiology, vol. 9. London: Elsevier; 1972. p. 183–255.

[14] Backus EA (1988). Sensory systems and behaviours which mediate hemipteran plant-feeding: a taxonomic overview. J Insect Physiol.

[15] Hattori M, Komatsu S, Noda H, Matsumoto Y (2015). Proteome analysis of watery saliva secreted by green rice leafhopper, Nephotettix cincticeps. PLoS One.

[16] Matsumoto Y, Suetsugu Y, Nakamura M, Hattori M (2014). Transcriptome analysis of the salivary glands of Nephotettix cincticeps (Uhler). J Insect Physiol.

[17] Huang H-J, Liu C-W, Huang X-H, Zhou X, Zhuo J-C, Zhang C-X, Bao Y-Y (2016). Screening and functional analyses of Nilaparvata lugens salivary proteome. J Proteome Res.

[18] Harmel N, Letocart E, Cherqui A, Giordanengo P, Mazzucchelli G, Guillonneau F, De Pauw E, Haubruge E, Francis F (2008). Identification of aphid salivary proteins: a proteomic investigation of Myzus persicae. Insect Mol Biol.

[19] Ji R, Yu H, Fu Q, Chen H, Ye W, Li S, Lou Y (2013). Comparative transcriptome analysis of salivary glands of two populations of rice brown planthopper, Nilaparvata lugens, that differ in virulence. PLoS One.

[20] Liu X, Zhou H, Zhao J, Hua H, He Y (2016). Identification of the secreted watery saliva proteins of the rice brown planthopper, Nilaparvata lugens (Stål) by transcriptome and shotgun LC–MS/MS approach. J Insect Physiol.

[21] Liu S, Bonning BC (2019). The principal salivary gland is the primary source of digestive enzymes in the saliva of the Brown Marmorated stink bug, *Halyomorpha halys*. Front Physiol.

[22] Li W, Zhao X, Yuan W, Wu K (2017). Activities of digestive enzymes in the omnivorous pest Apolygus lucorum (Hemiptera: Miridae). J Econ Entomol.

[23] Lomate PR, Bonning BC (2016). Distinct properties of proteases and nucleases in the gut, salivary gland and saliva of southern green stink bug, *Nezara viridula*. Sci Rep.

[24] Carolan JC, Fitzroy CI, Ashton PD, Douglas AE, Wilkinson TL (2009). The secreted salivary proteome of the pea aphid Acyrthosiphon pisum characterised by mass spectrometry. Proteomics.

[25] Cooper WR, Dillwith JW, Puterka GJ (2011). Comparisons of salivary proteins from five aphid (Hemiptera: Aphididae) species. Environ Entomol.

[26] Liu S, Lomate PR, Bonning BC (2018). Tissue-specific transcription of proteases and nucleases across the accessory salivary gland, principal salivary gland and gut of Nezara viridula. Insect Biochem Mol Biol.

[27] E-s S, Lin G-f, Liu S, Ma X-l, Chen M-f, Lin L, Wu S-q, Sha L, Liu Z-x, Hu X-h (2017). Identification of transcripts involved in digestion, detoxification and immune response from transcriptome of Empoasca vitis (Hemiptera: Cicadellidae) nymphs. Genomics.

[28] Taylor A (1993). Aminopeptidases: structure and function. FASEB J.

[29] Adang MJ. Insect aminopeptidase N. In: Handbook of proteolytic enzymes. London: Elsevier; 2004. p. 296–9.

[30] Akman Gündüz E, Douglas A (2008). Symbiotic bacteria enable insect to use a nutritionally inadequate diet. Proc R Soc B Biol Sci.

[31] Foissac X, Edwards M, Du J, Gatehouse A, Gatehouse J (2002). Putative protein digestion in a sap-sucking homopteran plant pest (rice brown plant hopper; Nilaparvata lugens: Delphacidae)--identification of trypsin-like and cathepsin B-like proteases. Insect Biochem Mol Biol.

[32] Wang P, Zhao J-Z, Rodrigo-Simón A, Kain W, Janmaat AF, Shelton AM, Ferré J, Myers J (2007). Mechanism of resistance to bacillus thuringiensis toxin Cry1Ac in a greenhouse population of the cabbage looper, *Trichoplusia ni*. Appl Environ Microbiol.

[33] Deraison C, Darboux I, Duportets L, Gorojankina T, Rahbé Y, Jouanin L (2004). Cloning and characterization of a gut-specific cathepsin L from the aphid *Aphis gossypii*. Insect Mol Biol.

[34] Law JH, Dunn PE, Kramer KJ (1977). Insect proteases and peptidases. Adv Enzymol Relat Areas Mol Biol.

[35] Macedo MR, Freire MGM (2011). Insect digestive enzymes as a target for pest control. Invertebr Surviv J.

[36] Kutsukake M, Shibao H, Nikoh N, Morioka M, Tamura T, Hoshino T, Ohgiya S, Fukatsu T (2004). Venomous protease of aphid soldier for colony defense. Proc Natl Acad Sci.

[37] Chacko SM, Thambi PT, Kuttan R, Nishigaki I (2010). Beneficial effects of green tea: a literature review. Chin Med.

[38] Serteyn L, Ponnet L, Backus EA, Francis F (2020). Characterization of electropenetrography waveforms for the invasive heteropteran pest, *Halyomorpha halys*, on *Vicia faba* leaves. Arthropod Plant Interact.

[39] Will T, Vilcinskas A (2015). The structural sheath protein of aphids is required for phloem feeding. Insect Biochem Mol Biol.

[40] Youn YN (1998). Electrically recorded feeding behavior of *Nephotettix cincticeps*. J Asia Pac Entomol.

[41] SoGAWA K (1967). Studies on the salivary glands of rice plant leafhoppers: II. Origins of the structural precursors of the sheath material. Appl Entomol Zool.

[42] Jing P, Bai S, Liu F (2013). Prelimitary research on electrical penetration graph (EPG) waveforms in relation to feeding behavior of *Laodelphax striatellus*. Chin J Appl Entomol.

[43] Ahmad A, Kaushik S, Ramamurthy V, Lakhanpaul S, Ramani R, Sharma K, Vidyarthi A (2012). Mouthparts and stylet penetration of the lac insect *Kerria lacca* (Kerr)(Hemiptera: Tachardiidae). Arthropod Struct De.

[44] Takeda Y. Studies on variations in genetic resources of tea in Japan and application to tea [*Camellia sinensis*] breeding. Bull Natl Inst Vegetable Tea Sci (Japan). 2002;1:97–180.

[45] Lawrence PK, Koundal KR (2002). Plant protease inhibitors in control of phytophagous insects. Electron J Biotechnol.

[46] Singh S, Singh A, Kumar S, Mittal P, Singh IK (2020). Protease inhibitors: recent advancement in its usage as a potential biocontrol agent for insect pest management. Insect Sci.

[47] Schlüter U, Benchabane M, Munger A, Kiggundu A, Vorster J, Goulet M-C, Cloutier C, Michaud D (2010). Recombinant protease inhibitors for herbivore pest control: a multitrophic perspective. J Exp Bot.

[48] Bradford MM (1976). A rapid and sensitive method for the quantitation of microgram quantities of protein utilizing the principle of protein-dye binding. Anal Biochem.

[49] Valaitis AP, Augustin S, Clancy KM (1999). Purification and characterization of the western spruce budworm larval midgut proteinases and comparison of gut activities of laboratory-reared and field-collected insects. Insect Biochem Mol Biol.

[50] Grabherr MG, Haas BJ, Yassour M, Levin JZ, Thompson DA, Amit I, Adiconis X, Fan L, Raychowdhury R, Zeng Q (2011). Trinity: reconstructing a full-length transcriptome without a genome from RNA-Seq data. Nat Biotechnol.

[51] Simão FA, Waterhouse RM, Ioannidis P, Kriventseva EV, Zdobnov EM. BUSCO: assessing genome assembly and annotation completeness with single-copy orthologs. Bioinformatics. 2015;31(19):3210–12. 10.1093/bioinformatics/btv351 26059717

[52] Li B, Dewey CN (2011). RSEM: accurate transcript quantification from RNA-Seq data with or without a reference genome. BMC Bioinformatics.

[53] Wu W, Liu H, Dong Y, Zhang Y, Wong S-M, Wang C, Zhou Y, Xu Q (2019). Determination of suitable RT-qPCR reference genes for studies of gene functions in *Laodelphax striatellus* (Fallén). Genes.

[54] Yang C, Pan H, Liu Y, Zhou X (2014). Selection of reference genes for expression analysis using quantitative real-time PCR in the pea aphid, *Acyrthosiphon pisum* (Harris) (Hemiptera, Aphidiae). PLoS One.

[55] Maroniche GA, Sagadín M, Mongelli VC, Truol GA, del Vas M (2011). Reference gene selection for gene expression studies using RT-qPCR in virus-infected planthoppers. Virol J.

[56] Mogilicherla K, Howell JL, Palli SR (2018). Improving RNAi in the Brown Marmorated stink bug: identification of target genes and reference genes for RT-qPCR. Sci Rep.

[57] Chen C, Chen H, Zhang Y, Thomas HR, Frank MH, He Y, Xia R: TBtools: an integrative toolkit developed for interactive analyses of big biological data. Molecular Plant. 2020;13(8):1194–1202. 10.1016/j.molp.2020.06.009 32585190

[58] Armenteros JJA, Tsirigos KD, Sønderby CK, Petersen TN, Winther O, Brunak S, von Heijne G, Nielsen H (2019). SignalP 5.0 improves signal peptide predictions using deep neural networks. Nat Biotechnol.

[59] Haas BJ, Papanicolaou A, Yassour M, Grabherr M, Blood PD, Bowden J, Couger MB, Eccles D, Li B, Lieber M, MacManes MD, Ott M, Orvis J, Pochet N, Strozzi F, Weeks N, Westerman R, William T, Dewey CN, Henschel R, LeDuc RD, Friedman N, Regev A (2013). De novo transcript sequence reconstruction from RNA-seq using the trinity platform for reference generation and analysis. Nat Protoc.

[60] Katoh K, Standley DM (2013). MAFFT multiple sequence alignment software version 7: improvements in performance and usability. Mol Biol Evol.

[61] Kalyaanamoorthy S, Minh BQ, Wong TK, von Haeseler A, Jermiin LS (2017). ModelFinder: fast model selection for accurate phylogenetic estimates. Nat Methods.

[62] Nguyen L-T, Schmidt HA, von Haeseler A, Minh BQ (2014). IQ-TREE: a fast and effective stochastic algorithm for estimating maximum-likelihood phylogenies. Mol Biol Evol.

[63] Hoang DT, Chernomor O, Von Haeseler A, Minh BQ, Vinh LS (2017). UFBoot2: improving the ultrafast bootstrap approximation. Mol Biol Evol.

