# Supplementary material for: Transcriptomic and proteomic analysis of putative digestive proteases in the salivary gland and gut of Empoasca (Matsumurasca) onukii Matsuda
Source: BMC Genomics. 2021 Apr 15;22:271. doi: 10.1186/s12864-021-07578-2 (PMC8048321; doi:10.1186/s12864-021-07578-2)
Supplement: Supplementary file 1 — Additional file 1. Additional tables. This file contains 2 additional tables [file 12864_2021_7578_MOESM1_ESM.pdf]

Table S1. Summary of *E. (M.) onukii* tissue specific transcriptome statistics

Tissue	Complete BUSCOs	Total No. of unigenes (≥200 bp)	Total nucleotides (bp)	Mean length (bp)	N50	Unigenes hit against NCBI Nr database	Proportion with unigenes hit against NCBI Nr database (%)	Encoding putative proteins (≥100aa)	Proportion encoding putative proteins ≥100aa (%)	No. of predicted proteins mapped by proteome peptides	% of predicted proteins identified in proteome (≥100 aa)
Salivary gland	90.6%	46,301	54,264,019	1,172	1,862	19,542	42.21	21,454	46.34	4457	21
Gut	88.8%	33,215	36,938,478	1,112	1,646	18,841	56.72	20,961	63.11	3784	18
Pooled* assembly	92.1%	138,056	136,388,783	988	1711	52,346	37.91	51,940	37.62	6674	13

* Reads from 3 replications in either midgut or salivary gland were combined for the Trinity assembly

Table S2. Number of putative digestion proteases identified from 5 hemipteran insects

	E. onukii		*A. pisum*		*L. striatellus*		*N. cincticeps*		*N. lugens*		*H. halys*	
	SG	Gut	SG	Gut	SG	Gut	SG	Gut	SG	Gut	SG*	Gut
Aminopeptidase	15	15	26	24	21	31	27	N/A	40	29	38	42
Carboxypeptidase	8	8	21	18	14	24	19	N/A	27	24	26	27
Dipeptidase	3	3	5	4	5	8	7	N/A	3	4	9	8
Aspartic protease	4	6	2	2	3	3	4	N/A	6	4	10	11
Cathepsin L-like	11	15	1	1	2	6	5	N/A	2	3	33	36
Cathepsin B-like	8	13	17	16	2	5	2	N/A	5	7	5	6
Serine protease	19	34	22	19	18	37	20	N/A	45	43	47	46

For detailed information about proteases in *E. onukii*, please refer to Additional File 3. For detailed information about proteases in other hemipteran insects, please refer to Additonal File 6.

*Proteases in the SG of *H. halys* include proteases in the primary salivary gland and the accessory salivary gland
