# Supplementary material for: Transcriptomic and proteomic analysis of putative digestive proteases in the salivary gland and gut of Empoasca (Matsumurasca) onukii Matsuda
Source: BMC Genomics. 2021 Apr 15;22:271. doi: 10.1186/s12864-021-07578-2 (PMC8048321; doi:10.1186/s12864-021-07578-2)
Supplement: Supplementary file 2 — Additional file 2. Additional figures. This file contains 4 additional figures [file 12864_2021_7578_MOESM2_ESM.pdf]

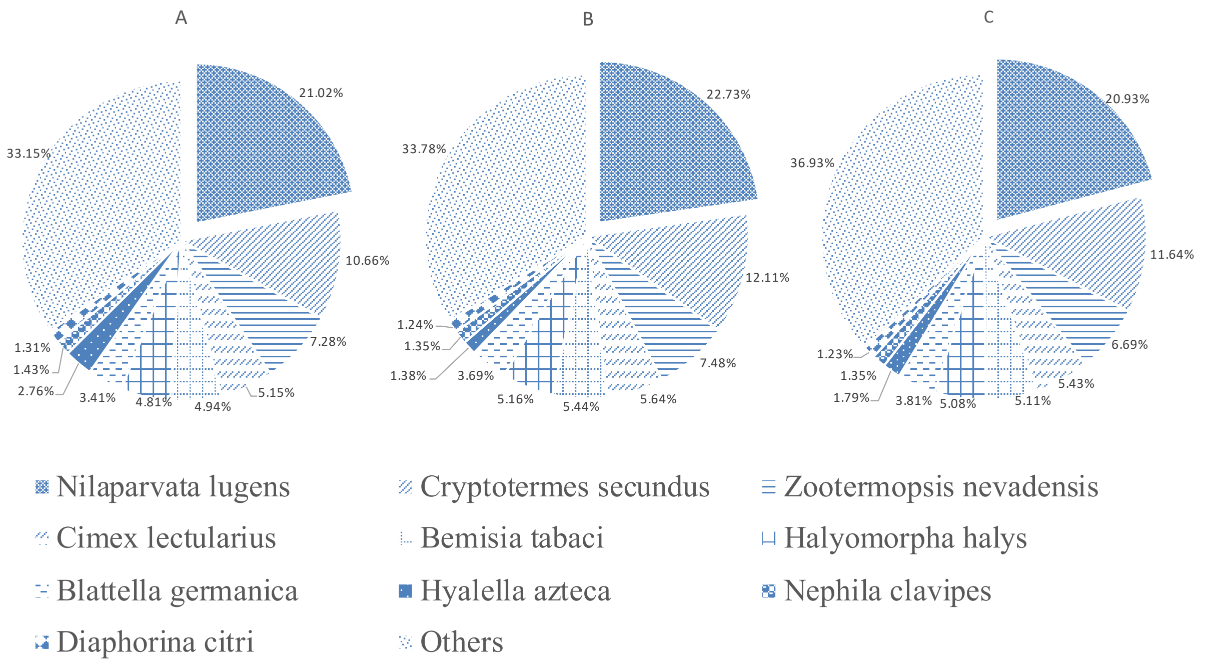

Figure S1. Species distribution of the best BLASTx hits in the nr database.
a: the assembled dataset from RNA-Seq reads of salivary glands; b: the assembled dataset from RNA-Seq reads of guts; c: the assembled dataset from pooled RNA-Seq reads of both tissues.

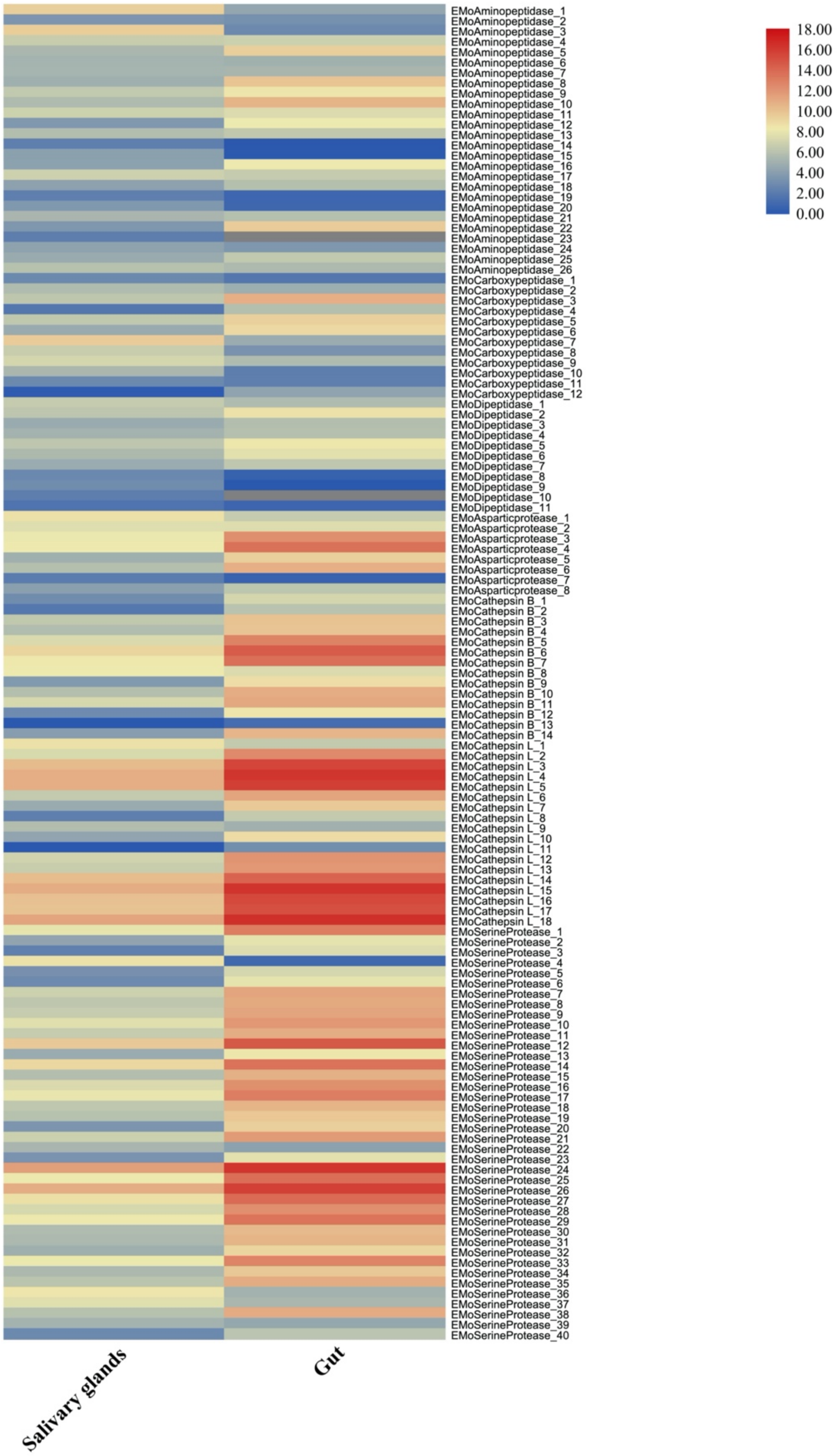

Figure S2. Heatmap presenting the transcript abundance of digestive proteases in the salivary glands and gut. Log10 of FPKM is presented by the color, ranging from blue (no expression) to red (high expression).

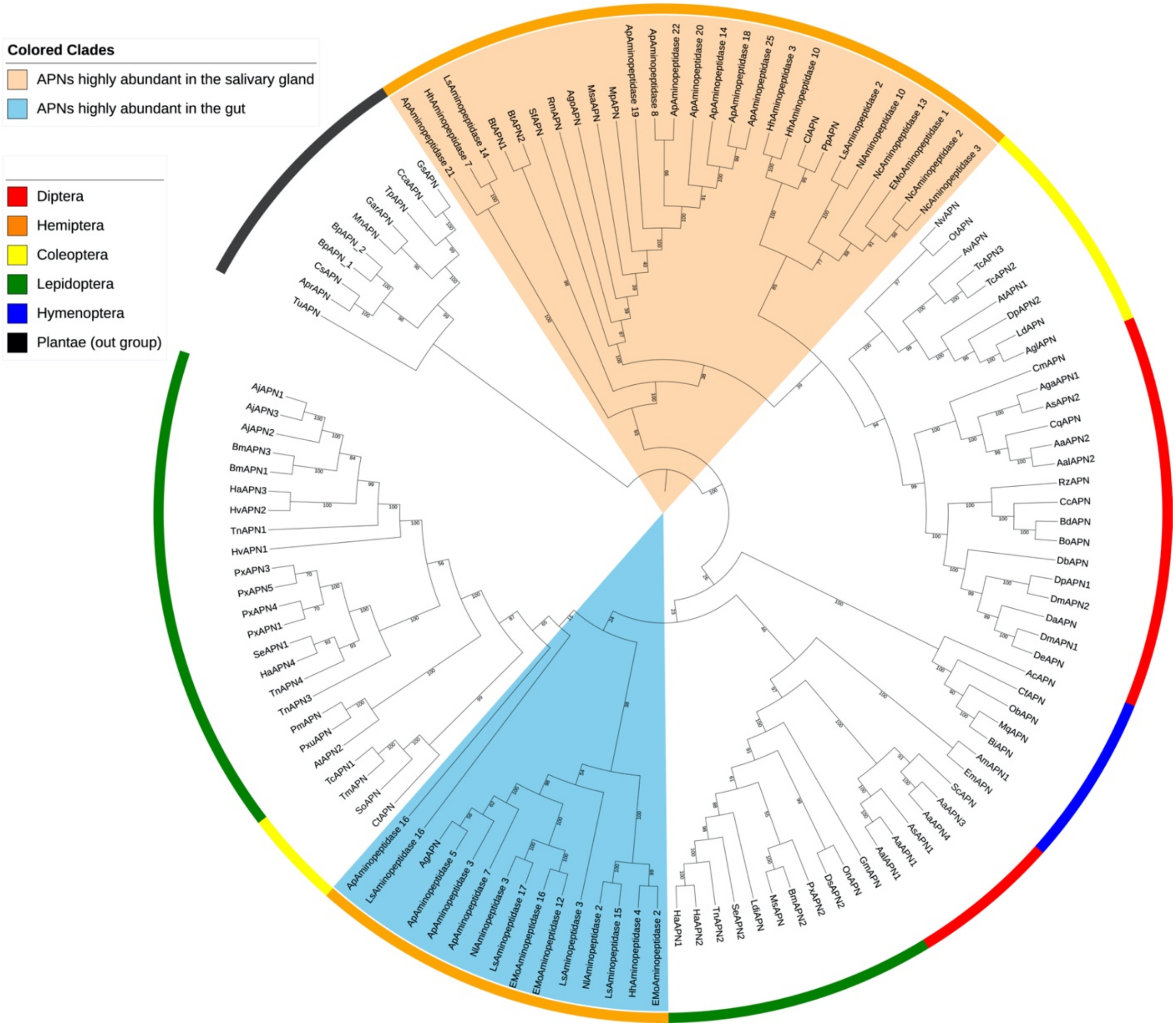

Figure S3. Phylogenetic tree containing aminopeptidases from multiple insect orders.

Protein sequences annotated by aminopeptidase N from *Nilaparvata lugens (Nl), Laodelphax striatellus (Ls), Nephotettix cincticeps (Nc), Acyrthosiphum pisim (Ap), Halyomorpha halys (Hh) and Empoasca (Matsumurasca) onukii (EMo)* were aligned with reference APNs downloaded from the NCBI (National Center of Biotechnology Information) database. Phylogenetic analysis was conducted through the maximum likelihood strategy. APNs derived from green plants were clustered as the outgroup. Accession numbers and information about the proteins are shown in Additional file 6. Information about putative aminopeptidases from *E. onukii* is shown in Additional file 3. The clades highlighted by orange and blue indicate hemipteran APNs highly abundant in the salivary glands and gut, respectively.

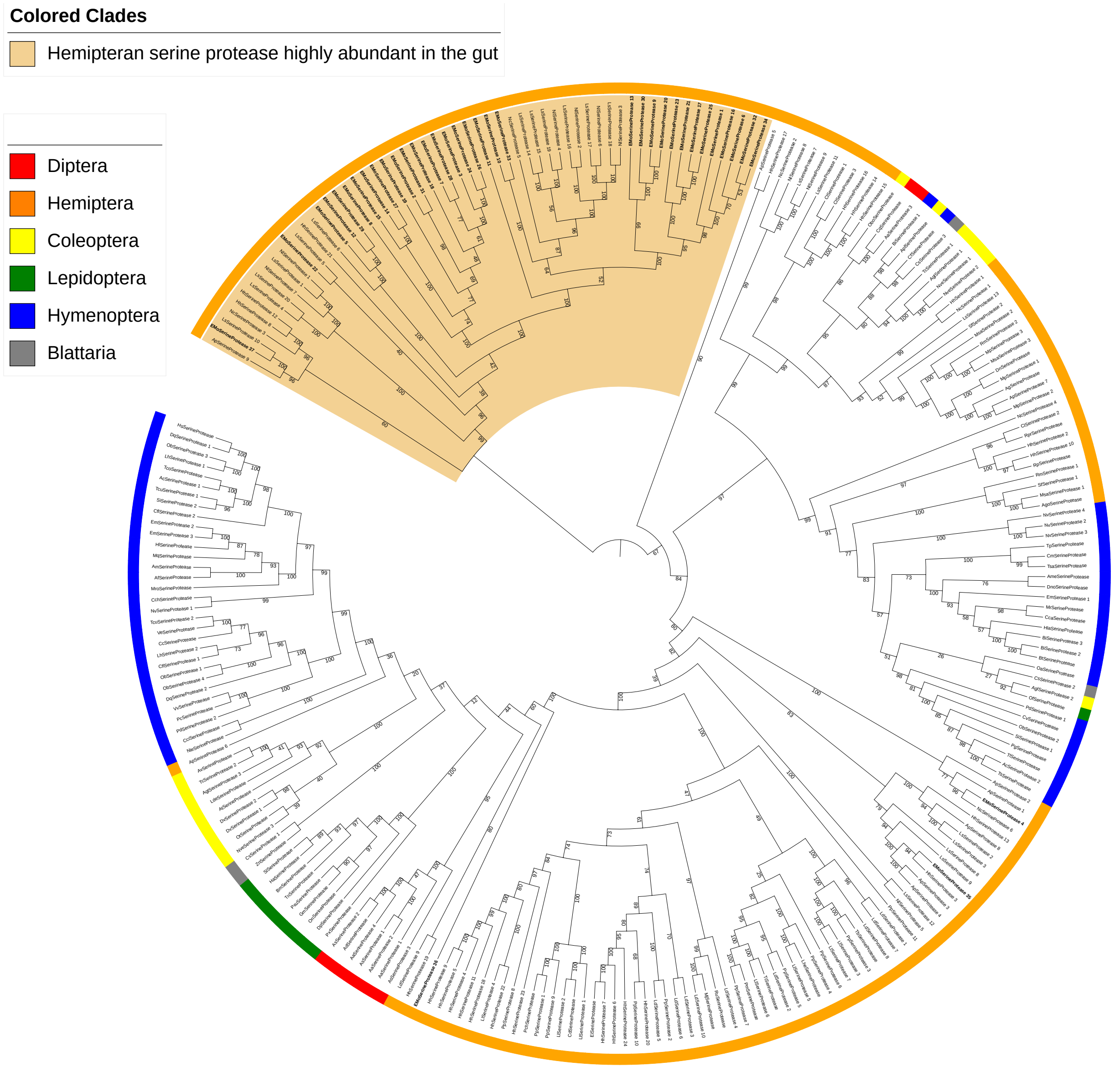

Figure S4. Phylogenetic tree containing serine protease-like proteins from multiple insect orders.
Protein sequences of annotated trypsin, chymotrypsin and elastase from *Nilaparvata lugens (Nl), Laodelphax striatellus (Ls), Nephotettix cincticeps (Nc), Acyrthosiphum pisim (Ap), Halyomorpha halys (Hh) and Empoasca (Matsumurasca) onukii (EMo)* were aligned with reference proteins annotated by venom serine protease in the NCBI (National Center of Biotechnology Information) database. Phylogenetic analysis was conducted through the maximum likelihood strategy. Accession numbers and information about the proteins from *Nilaparvata lugens, Laodelphax striatellus, Nephotettix cincticeps, Acyrthosiphum pisim, Halyomorpha halys* and other insects are shown in Additional file 6. Information about putative serine proteases from *E. onukii* is shown in Additional file 3. The clade highlighted by orange indicates hemipteran serine protease highly abundant in the gut.
